# Defining the Critical Elements of the Most Common Arthroscopic Procedures: A Consensus of Orthopaedic Sports Medicine Surgeons

**DOI:** 10.7759/cureus.4091

**Published:** 2019-02-19

**Authors:** David A Porter, Joseph L Laratta, Jamal N Shillingford, David Trofa, Hemant Reddy, John W Uribe, Gautam P Yagnik

**Affiliations:** 1 Orthopaedics, Baptist Health South Florida, Coral Gables, USA; 2 Orthopaedics, Norton Leatherman Spine Center, Louisville, USA; 3 Orthopedics, Norton Leatherman Spine Center, Louisville, USA; 4 Orthopedics, Columbia University Medical, New York, USA; 5 Orthopaedics, Northeast Ohio Medical University (NEOMED), Rootstown, USA

**Keywords:** sports medicine, arthroscopy, surgery, critical

## Abstract

Objective

To define the critical elements of common procedures in arthroscopic surgery.

Methods

A survey was administered to surgeons associated with the American Orthopaedic Society for Sports Medicine (AOSSM) to determine the critical elements for four common arthroscopic procedures: anterior cruciate ligament (ACL) reconstruction, knee arthroscopy with meniscal debridement or repair, rotator cuff repair (RCR), and capsulorrhaphy for anterior glenohumeral instability (Bankart repair). Respondents were asked which steps necessitated their direct supervision. The level of experience and practice demographics were also recorded.

Results

For all applicable procedures, patient positioning and closure were not considered critical steps. Establishing arthroscopic portals was critical for all procedures, except knee arthroscopy. Diagnostic arthroscopy was only critical in ACL reconstruction. Private practice surgeons considered every step of these common procedures to be critical elements. Less experienced surgeons were more likely to regard certain aspects of a procedure critical. Surgeons with >15 years of experience considered diagnostic arthroscopy critical to all procedures, whereas those with <15 years of experience did not. Unlike surgeons with a resident as first assist, surgeons with a physician assistant (PA) or nurse practitioner (NP) found every step of each procedure to be critical except closure and positioning.

Conclusion

Across all procedures, only patient positioning and closure were consistently regarded as non-critical elements. There were significant differences in responses according to experience and practice setting. Future research is necessary to determine the implications of these findings and guide the definition of the “critical portions” of surgery.

## Introduction

Every operation is a sequence of numerous, distinct steps that carry their own potential for complications. Academic teaching hospitals train resident surgeons by allowing them to assist in these operations and take an increasingly important role as both their knowledge and skills mature. The notion of “critical” steps of surgery, or those requiring the presence and direct supervision of the attending surgeon, has been implemented into nearly every document pertaining to surgery, from patient consent forms to national guidelines, and even billing clauses [1-2]. Recently, the Boston Globe’s investigation on the practice of concurrent surgeries drew controversy over “double booking” – the process of overlapping surgical cases booked under one attending surgeon in multiple rooms [1,3]. Questions of patient safety, ethical consent, health care costs, and medical education and training have arisen from this investigation despite the clear language in patient consent forms that reassures patients that an attending surgeon will be present for all “critical parts” of the procedure.

In April 2016, the American College of Surgeons’ (ACS') Statement of Principles was revised and distinguishes between “concurrent” and “overlapping” surgeries based on whether the “critical steps” of the two procedures occur simultaneously or sequentially [4]. This statement deems concurrent surgeries inappropriate, as they do not allow the presence of an attending surgeon during the “critical” steps of surgery occurring simultaneously. The Center for Medicare and Medicaid Services (CMS) has also declared that a supervising physician must be present for all “critical portions” of a procedure in order to qualify for reimbursement [2]. While the language in these consent forms is consistent across academic centers, this notion of “critical parts” of surgery still remains unclear, as it is open to various interpretations. To date, there has been no study establishing criteria for critical surgical steps in arthroscopic surgery, although it has been implied that such steps are “defined” for all common surgical procedures [5].

The purpose of the current study was to define the critical elements in four of the most common arthroscopic sports medicine procedures: anterior cruciate ligament (ACL) reconstruction, rotator cuff repair (RCR), knee arthroscopy, and anterior capsulorrhaphy with labral repair for glenohumeral instability (Bankart procedure). Herein, we report the results of a survey distributed to orthopedic sports medicine surgeons. We hypothesized that for each procedure, patient positioning, portal establishment, diagnostic arthroscopy, and closure would not be considered critical steps.

## Materials and methods

A web-based survey was developed by the authors at our institution and administered using Qualtrics software (Provo, UT, US), a survey platform for online data collection and analysis. The questionnaire consisted of 11 questions. The first seven questions were directed at surgeon demographics (years in practice, private versus academics, urban versus rural, geographic location, fellowship training, and first assistant). Each of the final four questions focused on a particular procedure, specifically ACL reconstruction, RCR, knee arthroscopy with meniscal treatment, and Bankart procedures. The order of the procedures was randomized for each respondent. Each of the procedures was separated into its constituent elements, including the positioning, portal placement, diagnostic arthroscopy, closure, and major steps in each respective procedure. The following statement headed each of these questions: “Which are the “critical” elements of surgery requiring your direct supervision in the operating room? Check ALL boxes that apply.” Respondents selected the portions of each procedure that necessitate their direct supervision in the operating room. If any of the major aspects of the procedure were selected by respondents, a sub-menu appeared with further questions. For the purpose of this study, a “critical” element of arthroscopic surgery was defined as a surgical step selected by the majority of respondents (>50%).

A link to the survey was emailed to 2016 members of the American Orthopaedic Society for Sports Medicine (AOSSM). Between August and September 2017, 343 physicians responded to the survey, corresponding to a response rate of 17% (343/2016).

Statistical analyses were performed with SPSS (IBM v24.0, Chicago, IL, US). An unadjusted univariate analysis was performed using independent sample t-tests for continuous data and Chi-squared or Fisher exact tests for categorical variables. Correlations between continuous variables were examined using the Pearson correlation coefficient test. Statistical significance was defined as p < 0.05.

## Results

A total of 343 surgeons responded to the survey. Responder demographics are presented in Table 1. The majority of respondents practiced in a major city (55.9%) with over 15 years of surgical experience (55%). Among respondents, the first assistant during surgery was a resident physician (30%), a physician assistant (PA) (45.9%), a nurse practitioner (NP) (4.1%), or other (20%). For all procedures, patient positioning and closure were not designated as critical elements. Diagnostic arthroscopy was not considered critical in any procedure, except for ACL reconstruction. The establishment of portals was a critical element of all procedures except knee arthroscopy.

**Table 1:** Responder demographics

All Responders (%)	
No. of Attending Surgeons	343 (17)
Years in Practice, n (%), N=340	
1-5 Years	30 (8.8)
6-10 Years	85 (25.0)
11-15 Years	38 (11.2)
15+ Years	187 (55.0)
Sports Medicine/Shoulder Fellowship Completed, n (%), N=341	
Sports Medicine	306 (89.7)
Shoulder	7 (2.1)
Neither	28 (8.2)
Practice Classification, n (%), N=341	
Academic	83 (24.3)
Private	183 (53.7)
Privademic	75 (22.0)
Practice Location, n (%), N=340	
Northeast	102 (30.0)
South	77 (22.6)
West	40 (11.8)
Central	85 (25.0)
Southwest	29 (8.5)
Outside of USA	7 (2.1)
Practice Location Classification, n (%), N=340	
Major City	190 (55.9)
Minor City	130 (38.9)
Rural	20 (5.9)
Cases Performed Each Year, n (%), N=341	
<100	6 (1.8)
100-250	68 (19.9)
250-500	186 (54.5)
500-700	57 (16.7)
>700	24 (7.0)
First Assistant During Surgery, n (%), N=340	
Resident (MD/DO)	102 (30.0)
Physician Assistant	156 (45.9)
Nurse Practitioner	14 (4.1)
Other	68 (20.0)

Among all respondents

For knee arthroscopy, meniscal debridement (66%), meniscal repair (95%), and chondral procedures (79%) were critical. When performing a meniscal debridement, determining which tears require meniscectomy (64%), identifying the proper amount of meniscus to resect (63.3%), and performing the meniscectomy (53%) were all considered critical. Additionally, all aspects of performing a meniscal repair were critical (Table 2). For chondral procedures, microfracture was deemed critical (76%) while performing a chondroplasty was not (47%).

**Table 2:** Critical steps of knee arthroscopy (*) signifies a critical element.

Overall Knee Arthroscopy Critical Elements, n (%), N=343	
Establishing Portals	154 (44.9)
Diagnostic Arthroscopy	166 (48.4)
Meniscal Debridement	225 (65.6) *
Meniscal Repair	327 (95.3) *
Chondral Procedures	271 (79.0) *
Closure	12 (3.5)
None	5 (1.5)
Positioning	47 (13.7)
Knee Arthroscopy Critical Establishing Portal Elements, n (%), N=343	
Anterior Portals	141 (41.1)
Posterior Portals for Meniscal Root Repair	143 (41.7)
Knee Arthroscopy Critical Meniscal Debridement Elements, n (%), N=343	
Identifying which Tears Need Meniscectomy vs Repair	218 (63.6) *
Identifying Amount of Meniscal Debridement	217 (63.3) *
Performing Meniscectomy with Shaver and/or Biter	180 (52.5) *
Knee Arthroscopy Critical Meniscal Repair Elements, n (%), N=343	
Establishing Technique (All inside/Outside In/ Inside Out)	284 (82.8) *
Approach if using Outside In/ Inside Out	268 (78.1) *
Identifying Location of Suture Placement	282 (82.2) *
All-Inside Repair	296 (86.3) *
Passing Sutures for Outside-in or Inside-out	287 (83.7) *
Tying Knots	204 (59.5) *
Knee Arthroscopy Critical Chondral Procedure Elements, n (%), N=343	
Microfracture	260 (75.8) *
Chondroplasty	161 (46.9)

For ACL reconstruction, all steps were critical except preparing the footprints, closing, and positioning (Table 3). With regard to autograft harvesting during ACL reconstruction, identifying (65%) and stripping the hamstring tendons (69%), selecting the size of bone-patella-bone (BTB) graft (63%), and harvesting the BTB graft with a microsagittal saw (81%) were critical steps, but preparation of the graft on the back table was not (20%). Performing a notchplasty and debriding the ACL ligament were not considered critical steps. Regarding tunnel placement, identifying the proper location (95%), holding the drill guide (68%), and drilling the tunnels (61%) were critical steps. Lastly, the steps of graft fixation were all critical, except for cycling the graft. This included shuttling the graft, determining the appropriate graft tension, and securing the graft with interference screws or aperture fixation.

**Table 3:** Critical steps of ACL reconstruction ACL - anterior cruciate ligament; BTB - bone-patella-bone (*) signifies a critical element.

Overall ACL Reconstruction Critical Elements, n (%), N=343	
Establishing Portals	180 (52.5) *
Graft Harvest	300 (87.5) *
Diagnostic Arthroscopy	179 (52.2) *
Debridement/Preparation of Footprints	189 (55.1) *
Tunnel Placement	335 (97.7) *
Graft Fixation	316 (92.1) *
Closing Patella Tendon/Closure	64 (18.7)
None	1 (0.3)
Positioning	96 (28.0)
ACL Reconstruction Critical Graft Harvest Elements, n (%), N=343	
Surgical Approach	166 (48.4)
Identifying Hamstring Tendons	224 (65.3) *
Stripping Tendons with Tendon Stripper	237 (69.1) *
BTB - Selecting Size of Patella Graft	215 (62.7) *
BTB - Using Microsagittal Saw to Harvest Bone Plugs	277 (80.8) *
Preparing Graft on Back Table	67 (19.5)
ACL Reconstruction Critical Debridement/Footprint Preparation Elements, n (%), N=343	
Notchplasty	142 (41.4)
Debriding Footprints/ Removing ACL Stump	149 (43.4)
ACL Reconstruction Critical Tunnel Placement Elements, n (%), N=343	
Identifying Tunnel Position	324 (94.5) *
Holding Guide for Guide Pins	232 (67.6) *
Drilling Tunnels	210 (61.2) *
ACL Reconstruction Critical Graft Fixation Elements, n (%), N=343	
Graft Passage/Shuttling	249 (72.6) *
Cycling Graft	139 (40.5)
Determining Appropriate Graft Tension	260 (75.8) *
Placing Interference Screws / Aperture Fixation	298 (86.9) *

For rotator cuff repair, establishing portals (54%), preparing the footprint (64%), and repairing the tendon (94%) were critical. Positioning, diagnostic arthroscopy, subacromial bursectomy, and closure were found to be non-critical steps (Table 4). Further evaluation found that all aspects of repairing the tendon were critical, including identifying suture placement, passing sutures, identifying the number and location of anchors, determining single versus double row, and tying suture knots.

**Table 4:** Critical steps of rotator cuff repair (*) signifies a critical element.

Overall Rotator Cuff Repair Critical Elements, n (%), N=343	
Establishing Portals	185 (53.9) *
Diagnostic Arthroscopy	171 (49.9)
Subacromial Bursectomy	149 (43.4)
Preparing Footprint	221 (64.4) *
Repairing Tendon	323 (94.2) *
Closure	13 (3.8)
None	2 (0.6)
Positioning	96 (28.0)
Rotator Cuff Repair Critical Establishing Portal Elements, n (%), N=343	
Portals in Lateral Decubitus Position	152 (44.3)
Portals in Beach Chair Position	133 (38.8)
Rotator Cuff Repair Critical Subacromial Bursectomy Elements, n (%), N=343	
Bursectomy	129 (37.6)
Acromioplasty	143 (41.7)
Tear Assessment	147 (42.9)
Rotator Cuff Repair Critical Footprint Preparation Elements, n (%), N=343	
Using Shaver/Burr to Decorticate Humeral Footprint	176 (51.3) *
Debriding Tendon Edges	163 (47.5)
Tendon Mobilization	215 (62.7) *
Marginal Convergence	217 (63.3) *
Interval Slide	206 (60.1) *
Rotator Cuff Repair Critical Tendon Repair Elements, n (%), N=343	
Identifying Location of Suture Placement	298 (86.9) *
Passing Sutures with Suture Passer	261 (76.1) *
Passing Sutures with Suture Lasso	212 (61.8) *
Identifying Number of Anchors	285 (83.1) *
Identifying Location of Anchors	306 (89.2) *
Determining Singer vs Double Row	274 (79.9) *
Tying Suture Knots	258 (75.2) *

For arthroscopic anterior capsulorrhaphy with labral repair (or Bankart procedure), the critical elements were the establishment of portals (64%), glenoid preparation (80%), passing sutures (90%), and placing anchors (93%) (Table 5). Within glenoid preparation, both the mobilization of the labrum and decorticating the glenoid neck were critical. Passing sutures, the selection of the number and placement of anchors, drilling for anchors, and tying suture knots were all found to be critical. Similar to arthroscopic rotator cuff repair, diagnostic arthroscopy and closure were not critical.

**Table 5:** Critical steps of anterior capsulorrhaphy with labral repair (Bankart procedure) (*) signifies a critical element.

Overall Capsulorrhaphy, Anterior; with Labral Repair (i.e. Bankart Procedure) Critical Elements, n (%), N=343	
Establishing Portals	221 (64.4) *
Diagnostic Arthroscopy	169 (49.3)
Preparing Glenoid	274 (79.9) *
Passing Sutures	309 (90.1) *
Placing Anchors	320 (93.3) *
Closure	6 (1.7)
Positioning	107 (31.2)
Bankart Repair Critical Establishing Portals Elements, n (%), N=343	
Portals in Lateral Decubitus Position	185 (53.9) *
Portals in Beach Chair Position	139 (40.5)
Bankart Repair Critical Glenoid Preparation Elements, n (%), N=343	
Mobilizing Labrum	271 (79.0) *
Decorticating Glenoid Neck	240 (70.0) *
Bankart Repair Critical Passing Sutures Elements, n (%), N=343	
Passing Sutures at 3 o'clock Position	225 (65.6) *
Passing Sutures at 5-6 o'clock Position	307 (89.5) *
Bankart Repair Critical Placing Anchors Elements, n (%), N=343	
Selecting Number of Anchors	276 (80.5) *
Selecting Location of Anchors	308 (89.8) *
Using Knotless Anchors	226 (65.9) *
Using Traditional Anchors	228 (66.5) *
Drilling Anchors	274 (79.9) *
Tying Suture Knots	255 (74.3) *

Private versus academic

For knee arthroscopy, private practice surgeons rated all aspects of knee arthroscopy except closure and positioning to be critical. Academic surgeons, on the other hand, did not find portal establishment (p<0.0001) or diagnostic arthroscopy (p<0.0001) to be critical. With regards to chondral procedures, private surgeons found chondroplasty to be a critical element while academic surgeons did not (p=0.007). For ACL reconstruction, private surgeons rated portal establishment (p<0.0001), diagnostic arthroscopy (p<0.0001), and debridement of footprint/ACL (p=0.012) to be critical while academic surgeons did not.

For RCR, private surgeons rated portal establishment (p<0.0001), diagnostic arthroscopy (p<0.0001), and subacromial decompression (p=0.006) as critical steps while academic surgeons did not. For the Bankart procedure, private surgeons rated diagnostic arthroscopy (p<0.0001) and portal establishment (p<0.0001) as critical while academic surgeons did not (Table 6).

**Table 6:** Differences according to setting (academic vs. private) ACL - anterior cruciate ligament (*) signifies a critical element.

	Academic	Private	P-value
Number of Responders	83	183	-
Overall ACL Reconstruction Critical Elements, n (%), N=266			
Establishing Portals	28 (33.7)	121 (66.1)	<0.0001
Diagnostic Arthroscopy	28 (33.7)	122 (66.7)	<0.0001
Debridement/Preparation of Footprints	40 (48.2)	118 (64.5)	0.012
Closing Patella Tendon/Closure	4 (4.8)	44 (24.0)	<0.0001
Positioning	17 (20.5)	62 (33.9)	0.027
ACL Reconstruction Critical Graft Harvest Elements, n (%), N=266			
Surgical Approach	27 (32.5)	110 (60.1)	<0.0001
ACL Reconstruction Critical Debridement/Footprint Preparation Elements, n (%), N=266			
Notchplasty	28 (33.7)	89 (48.6)	0.023
Debriding Footprints/ Removing ACL Stump	31 (37.3)	94 (51.4)	0.034
ACL Reconstruction Critical Tunnel Placement Elements, n (%), N=266			
Identifying Tunnel Position	75 (90.4)	178 (97.3)	0.027
Overall Knee Arthroscopy Critical Elements, n (%), N=266			
Establishing Portals	20 (24.1)	102 (55.7)	<0.0001
Diagnostic Arthroscopy	24 (28.9)	109 (59.6)	<0.0001
Knee Arthroscopy Critical Establishing Portal Elements, n (%), N=266			
Anterior Portals	18 (21.7)	96 (52.5)	<0.0001
Posterior Portals for Meniscal Root Repair	18 (21.7)	96 (52.5)	<0.0001
Knee Arthroscopy Critical Chondral Procedure Elements, n (%), N=266			
Chondroplasty	31 (37.3)	101 (55.2)	0.007
Overall Rotator Cuff Repair Critical Elements, n (%), N=266			
Establishing Portals	27 (32.5)	123 (67.2)	<0.0001
Diagnostic Arthroscopy	23 (27.7)	114 (62.3)	<0.0001
Subacromial Bursectomy	28 (33.7)	95 (51.9)	0.006
Positioning	15 (18.1)	62 (33.9)	0.008
Rotator Cuff Repair Critical Establishing Portal Elements, n (%), N=266			
Portals in Lateral Decubitus Position	24 (28.9)	102 (55.7)	<0.0001
Portals in Beach Chair Position	20 (24.1)	87 (47.5)	<0.0001
Rotator Cuff Repair Critical Subacromial Bursectomy Elements, n (%), N=266			
Bursectomy	20 (24.1)	89 (48.6)	<0.0001
Acromioplasty	25 (30.1)	93 (50.8)	0.002
Tear Assessment	28 (33.7)	93 (50.8)	0.01
Rotator Cuff Repair Critical Footprint Preparation Elements, n (%), N=266			
Using Shaver/Burr to Decorticate Humeral Footprint	34 (41.0)	108 (59.0)	0.006
Debriding Tendon Edges	32 (38.6)	100 (54.6)	0.015
Overall Capsulorrhaphy, Anterior; with Labral Repair (i.e. Bankart Procedure) Critical Elements, n (%), N=266			
Establishing Portals	39 (47.0)	135 (73.8)	<0.0001
Diagnostic Arthroscopy	24 (28.9)	112 (61.2)	<0.0001
Bankart Repair Critical Establishing Portals Elements, n (%), N=266			
Portals in Lateral Decubitus Position	33 (39.8)	115 (62.8)	<0.0001
Portals in Beach Chair Position	23 (27.7)	85 (46.4)	0.004

Years in practice

Based on years of experience, surgeons with less experience (<15 years) did not rate portal establishment, diagnostic arthroscopy, or ligament debridement/notchplasty as critical ACL reconstruction steps while older surgeons did. Similar results were obtained for knee arthroscopy, with portal establishment and diagnostic arthroscopy regarded as non-critical by younger surgeons but critical by those with greater than 15 years of experience. Surgeons with less experience did not regard chondroplasty and meniscal debridement as critical, while the more experienced surgeons did (p=0.032, p= 0.004, respectively). For RCR, experienced surgeons considered decorticating footprint (p= 0.38) and debriding tendon edges (p=0.183) to be critical while less experienced surgeons did not, however, the difference did not meet significance. All surgeons regardless of experience considered every aspect of the tendon repair (passing sutures, identifying the location of anchors, placing anchors, tying knots) to be critical. When comparing across surgeons for the Bankart procedure, closure and patient positioning were similarly regarded as non-critical aspects of the procedure. Younger surgeons, however, did not consider diagnostic arthroscopy to be critical (47% vs. 50%, p=0.535). Both cohorts regarded all sub-steps of this procedure to be critical and there were no differences between them.

First assistant

When comparing across years in practice, there was no difference in first assistant utilization. First assistants included residents, physician assistants (PAs), and nurse practitioners (NPs). Unlike surgeons with a resident as first assist, surgeons with a PA or NP found every step of each procedure to be critical except closure and positioning (Table 7). For all procedures, when a resident was first assistant, positioning, portal establishment, diagnostic arthroscopy, and closure were not found to be critical. For ACL reconstruction, when a resident was the first assistant, the only critical steps were graft harvest, tunnel placement, and graft fixation. For knee arthroscopy, chondroplasty was not found to be critical when a resident was first assistant. For RCR, tendon repair was the only critical step.

**Table 7:** Differences according to first assistant PA - physician assistant, NP - nurse practitioner, ACL - anterior cruciate ligament

	Resident First Assist	PA/NP	P-value
Number of Responders	102	169	-
Overall ACL Reconstruction Critical Elements, n (%), N=343			
Establishing Portals	33 (32.4)	105 (61.8)	<0.0001
Diagnostic Arthroscopy	30 (29.4)	110 (64.7)	<0.0001
Debridement/Preparation of Footprints	39 (38.2)	110 (64.7)	<0.0001
Closing Patella Tendon/Closure	6 (5.9)	35 (20.6)	0.001
ACL Reconstruction Critical Graft Harvest Elements, n (%), N=272			
Surgical Approach	29 (28.4)	100 (58.8)	<0.0001
ACL Reconstruction Critical Debridement/Footprint Preparation Elements, n (%), N=272			
Notchplasty	23 (22.5)	88 (51.8)	<0.0001
Debriding Footprints/ Removing ACL Stump	29 (28.4)	87 (51.2)	<0.0001
ACL Reconstruction Critical Tunnel Placement Elements, n (%), N=272			
Identifying Tunnel Position	91 (89.2)	169 (99.4)	<0.0001
Overall Knee Arthroscopy Critical Elements, n (%), N=272			
Establishing Portals	25 (24.5)	93 (54.7)	<0.0001
Diagnostic Arthroscopy	28 (27.5)	103 (60.6)	<0.0001
Meniscal Debridement	60 (58.8)	124 (72.9)	0.016
Positioning	6 (5.9)	23 (13.5)	0.048
Knee Arthroscopy Critical Establishing Portal Elements, n (%), N=272			
Anterior Portals	20 (19.6)	87 (51.2)	<0.0001
Posterior Portals for Meniscal Root Repair	21 (20.6)	87 (51.2)	<0.0001
Knee Arthroscopy Critical Meniscal Debridement Elements, n (%), N=272			
Identifying which Tears Need Meniscectomy vs Repair	56 (54.9)	122 (71.8)	0.005
Identifying Amount of Meniscal Debridement	58 (56.9)	119 (70.0)	0.028
Performing Meniscectomy with Shaver and/or Biter	41 (40.2)	103 (60.6)	0.001
Knee Arthroscopy Critical Meniscal Repair Elements, n (%), N=272			
Establishing Technique (All inside/Outside In/ Inside Out)	77 (75.5)	148 (87.1)	0.015
Approach if using Outside In/ Inside Out	75 (73.5)	143 (84.1)	0.034
Tying Knots	54 (52.9)	111 (65.3)	0.043
Knee Arthroscopy Critical Chondral Procedure Elements, n (%), N=272			
Chondroplasty	35 (34.3)	92 (54.1)	0.002
Overall Rotator Cuff Repair Critical Elements, n (%), N=272			
Establishing Portals	31 (30.4)	110 (64.7)	<0.0001
Diagnostic Arthroscopy	27 (26.5)	108 (63.5)	<0.0001
Subacromial Bursectomy	28 (27.5)	91 (53.5)	<0.0001
Preparing Footprint	57 (55.9)	118 (69.4)	0.024
Rotator Cuff Repair Critical Establishing Portal Elements, n (%), N=272			
Portals in Lateral Decubitus Position	23 (22.5)	92 (54.1)	<0.0001
Portals in Beach Chair Position	22 (21.6)	79 (46.5)	<0.0001
Rotator Cuff Repair Critical Subacromial Bursectomy Elements, n (%), N=272			
Bursectomy	18 (17.6)	83 (48.8)	<0.0001
Acromioplasty	23 (22.5)	90 (52.9)	<0.0001
Tear Assessment	28 (27.5)	89 (52.4)	<0.0001
Rotator Cuff Repair Critical Footprint Preparation Elements, n (%), N=272			
Using Shaver/Burr to Decorticate Humeral Footprint	39 (38.2)	98 (57.6)	0.002
Debriding Tendon Edges	36 (35.3)	90 (52.9)	0.005
Tendong Mobilization	55 (53.9)	115 (67.6)	0.024
Marginal Convergence	55 (53.9)	116 (68.2)	0.018
Interval Slide	51 (50.0)	112 (65.9)	0.01
Rotator Cuff Repair Critical Tendon Repair Elements, n (%), N=272			
Identifying Location of Suture Placement	84 (82.4)	154 (90.6)	0.047
Identifying Location of Anchors	88 (86.3)	160 (94.1)	0.027
Overall Capsulorrhaphy, Anterior; with Labral Repair (i.e. Bankart Procedure) Critical Elements, n (%), N=272			
Establishing Portals	50 (49.0)	124 (72.9)	<0.0001
Diagnostic Arthroscopy	26 (25.5)	108 (63.5)	<0.0001
Bankart Repair Critical Establishing Portals Elements, n (%), N=272			
Portals in Lateral Decubitus Position	37 (36.3)	108 (63.5)	<0.0001
Portals in Beach Chair Position	31 (30.4)	77 (45.3)	0.015
Bankart Repair Critical Glenoid Preparation Elements, n (%), N=272			
Decorticating Glenoid Neck	64 (62.7)	128 (75.3)	0.028

## Discussion

Every surgical procedure is a series of steps, some more critical than others. Some steps of a procedure are so important that any misstep or lack of guidance may expose the patient to undue harm. Despite their crucial role in surgical training, billing and the ethicality of concurrent surgery, the “critical steps” of arthroscopy procedures have not yet been clearly defined and interpretation has fallen in the hands of the surgeon. The current study aims to reach a greater consensus of which steps are generally deemed critical by surgeons and to identify how this may vary according to certain demographic parameters. The elucidation of critical elements for surgical procedures has potential ramifications on surgical education, concurrent surgery, surgical billing, and medical ethics.

Recent press reports have suggested that there is an increase in adverse patient outcomes and longer procedure times when an attending surgeon is operating in two different surgical suites [1]. The concept of concurrent surgeries in orthopedic literature is limited; however, a recent analysis of overlapping surgery in the ambulatory setting has been described. Zhang et al. performed a retrospective review over a three-year period and found that 68% of cases were concurrent while 32% were not [6]. They found no difference in the postoperative complication rate between the cohorts (1.1% vs. 1.3%, p=0.811). They also concluded that overlapping surgery yields an equivalent operating time in an ambulatory setting [6]. National registry data from the American College of Surgeons in greater than 20,000 knee and shoulder arthroscopic cases has shown these procedures to be inherently safe, with a 30-day complication rate of 1.6% and 0.99%, respectively [7-8].

We report that for the most common arthroscopic sports medicine surgeries, there was considerable variability in the elements deemed “critical” by the surveyed respondents. Patient positioning and closure were not deemed critical steps among all procedures. Diagnostic arthroscopy was only critical when performing ACL reconstruction. Interestingly, the establishment of arthroscopic portals was viewed as critical for all procedures except knee arthroscopy.

We found significant differences between academic and private practice surgeons, suggesting that the notion of “critical element” may be influenced by the surgical setting. Notably, nearly all steps in all four procedures were considered critical by private practice surgeons. Often, surgeons practicing in a private setting lack highly trained surgical assistants, such as residents and fellows, who are qualified and capable of performing a number of operative steps independently. As a result, nearly every step of a surgical procedure in a private practice requires the direct supervision of the surgeon.

Younger surgeons, defined as those with less than 15 years of practice, were significantly less likely to consider diagnostic arthroscopy and portal establishment as critical steps of the procedure. The difference is likely, in part, due to a comfort level with arthroscopy cases since nearly 98% of the younger respondents had completed a sports medicine fellowship compared to only 82% of the more experienced surgeons (p<0.01).

Although completely novel in the sports medicine literature, there are limitations to the current study. First, the study is limited by the response rate. The survey was distributed to over 2,000 surgeons; however, only 17% responded to the survey. One reason for an imperfect response rate may be that there is no way of identifying how many surgeons received the email. Some of these emails may have been processed as spam or the email listed is not the primary email used by the surgeon. Overall, establishing a consensus regarding the “critical” elements of surgery may necessitate a more robust sample size. Lastly, a potential limitation is that there are likely other factors that influence how surgeons classify different steps of surgery. Patient characteristics, including body weight, comorbid conditions, anatomic variations, and prior arthroscopic procedures, may significantly influence a surgeon’s perception of the surgical procedure.

## Conclusions

The notion of “critical” is used colloquially by the medical community in the form of national guidelines, consent forms, and reimbursement regulations. Until now, there have not been any attempts to define critical steps of arthroscopic sports medicine procedures. For four of the most common arthroscopic sports medicine procedures, elements that were not regarded as critical routinely included positioning, the establishment of portals, and closure. However, given the variability based on surgical subspecialty, surgical setting, and surgeon experience, it is difficult to reach a general consensus and standardized definitions of “critical” elements should be established by professional sports medicine societies.
